# Comparative Transcriptome Analysis of Genes Involved in Anthocyanin Biosynthesis in the Red and Yellow Fruits of Sweet Cherry (*Prunus avium* L.)

**DOI:** 10.1371/journal.pone.0121164

**Published:** 2015-03-23

**Authors:** Hairong Wei, Xin Chen, Xiaojuan Zong, Huairui Shu, Dongsheng Gao, Qingzhong Liu

**Affiliations:** 1 College of Horticulture Science and Engineering, Shandong Agricultural University, Tai’an, Shandong 271018, China; 2 Key Laboratory for Fruit Biotechnology Breeding of Shandong, Shandong Institute of Pomology, Shandong Academy of Agricultural Sciences, Tai’an, Shandong 271000, China; NARO Institute of Fruit Tree Science, JAPAN

## Abstract

**Background:**

Fruit color is one of the most important economic traits of the sweet cherry (*Prunus avium* L.). The red coloration of sweet cherry fruit is mainly attributed to anthocyanins. However, limited information is available regarding the molecular mechanisms underlying anthocyanin biosynthesis and its regulation in sweet cherry.

**Methodology/Principal Findings:**

In this study, a reference transcriptome of *P*. *avium* L. was sequenced and annotated to identify the transcriptional determinants of fruit color. Normalized cDNA libraries from red and yellow fruits were sequenced using the next-generation Illumina/Solexa sequencing platform and *de novo* assembly. Over 66 million high-quality reads were assembled into 43,128 unigenes using a combined assembly strategy. Then a total of 22,452 unigenes were compared to public databases using homology searches, and 20,095 of these unigenes were annotated in the Nr protein database. Furthermore, transcriptome differences between the four stages of fruit ripening were analyzed using Illumina digital gene expression (DGE) profiling. Biological pathway analysis revealed that 72 unigenes were involved in anthocyanin biosynthesis. The expression patterns of unigenes encoding phenylalanine ammonia-lyase (PAL), 4-coumarate-CoA ligase (4CL), chalcone synthase (CHS), chalcone isomerase (CHI), flavanone 3-hydroxylase (F3H), flavanone 3’-hydroxylase (F3’H), dihydroflavonol 4-reductase (DFR), anthocyanidin synthase (ANS) and UDP glucose: flavonol 3-O-glucosyltransferase (UFGT) during fruit ripening differed between red and yellow fruit. In addition, we identified some transcription factor families (such as MYB, bHLH and WD40) that may control anthocyanin biosynthesis. We confirmed the altered expression levels of eighteen unigenes that encode anthocyanin biosynthetic enzymes and transcription factors using quantitative real-time PCR (qRT-PCR).

**Conclusions/Significance:**

The obtained sweet cherry transcriptome and DGE profiling data provide comprehensive gene expression information that lends insights into the molecular mechanisms underlying anthocyanin biosynthesis. These results will provide a platform for further functional genomic research on this fruit crop.

## Introduction

Sweet cherry (*P*. *avium* L.) is one of the most popular fresh fruits grown in temperate regions worldwide because of its appealing color, delicious taste and nutritional value. The color of the fruit ranges from dark red to pale yellow and is one of the primary exterior quality and economic characteristics, along with fruit weight and sweetness, that influences consumers’ buying decisions [1]. Fruit color is also the most important indicator of the quality and maturity of fresh cherries [2]. The color of cherries has been attributed to the accumulation of anthocyanins, and differences in the types and levels of anthocyanins vary between cherries of different colors [3, 4, 5].

Anthocyanins are secondary metabolites that not only play a significant role in pigmentation but also have antioxidant [6, 7] and anti-tumor functions [8, 9], protect against coronary heart disease and help defend against pathogens and ultraviolet radiation [6]. The accumulation and distribution of anthocyanins are governed by metabolic networks that are regulated by genetic and environmental conditions; they are also strongly correlated with the expression of structural and regulatory genes [10]. In model plants such as *Arabidopsis thaliana*, the structural genes in the anthocyanin biosynthetic pathway are divided into two groups designated as “early” or “late”. The “early” biosynthetic genes appear to be coordinately regulated and encode enzymes that function in the beginning steps of the biosynthetic pathway, such as CHS and CHI. The “late” biosynthetic genes are expressed during the later stages of the biosynthetic pathway, such as DFR, ANS and others [11]. During blooming and fruit ripening, these genes exhibit different expression patterns in different species [12]. The transcriptional regulation of structural genes plays an important role in the anthocyanin biosynthetic pathway. The transcriptional regulators for anthocyanin biosynthesis include MYB proteins, bHLH (basic helix-loop-helix) proteins and WD40 proteins [13, 14]. The combinations of and interactions between the MYB, bHLH and WD40 transcription factors mediate the regulation of the anthocyanin biosynthetic pathway [15]. The structural genes and transcription factors that are involved in the anthocyanin biosynthetic pathway have been identified through biochemical and genetic analyses in several fruit trees, including strawberry, apple, and grape [16, 17, 18, 19].

In recent years, more and more researchers have focused on the genetic control of skin color in sweet cherry. For example, Suneth et al. reported a QTL analysis and candidate gene mapping for skin and flesh color in sweet cherry fruit [20]. Wang et al. analyzed *ANS* and *CHS* expression levels in the red fruit cultivar ‘Stella’ and the bicolored cultivar ‘Rainier’ [21]. The expression levels of six genes involved in anthocyanin biosynthesis have previously been studied in the red fruit cultivar ‘Hongdeng’ and the bicolored cultivar ‘Caihong’ using qRT-PCR analysis [22]. However, the precise hierarchical organization of the global network that is involved in anthocyanin biosynthesis has not been defined.

More recently, transcriptome analyses based on deep sequencing have been used for gene discovery, the analysis of specific transcripts, and the estimation of overall gene expression at different developmental stages and in different tissues. Next-generation sequencing and DGE profiling are cost-effective choices for characterizing non-model organisms without a reference genome [23, 24]. The anthocyanin biosynthetic pathway and the expression of related genes have been studied through global transcriptome methods, uncovering a wealth of anthocyanin biosynthesis regulatory genes in important fruit crops such as grape [25], blood orange [26], blueberry [27], Chinese bayberry [28], pomegranate [29], and black raspberry [30]. A transcriptome analysis of sweet cherry has been reported, but that study was focused on cuticle deposition [31].

Pure yellow cultivars are rare germplasm in sweet cherry breeding, and they are precious materials for the study of anthocyanin biosynthesis. In the present study, the pure yellow cultivar ‘13–33’ and the red cultivar ‘Tieton’ were used as the experimental material to establish a database of transcriptome sequences of sweet cherry fruit using Illumina transcriptome sequencing. The transcriptome database was used as reference data to identify candidate genes involved in the biosynthesis of anthocyanins. Based on this, we used DGE profile analysis to compare the transcripts involved in the biosynthesis of the anthocyanins that exist in the red and yellow cultivars. The transcriptome sequences and gene expression profiles provide a solid foundation for functional genomic studies on sweet cherry in the future and will facilitate a better understanding of the molecular mechanisms of anthocyanin biosynthesis.

## Materials and Methods

### Plant material and determination of total anthocyanins

**Fig 1:**
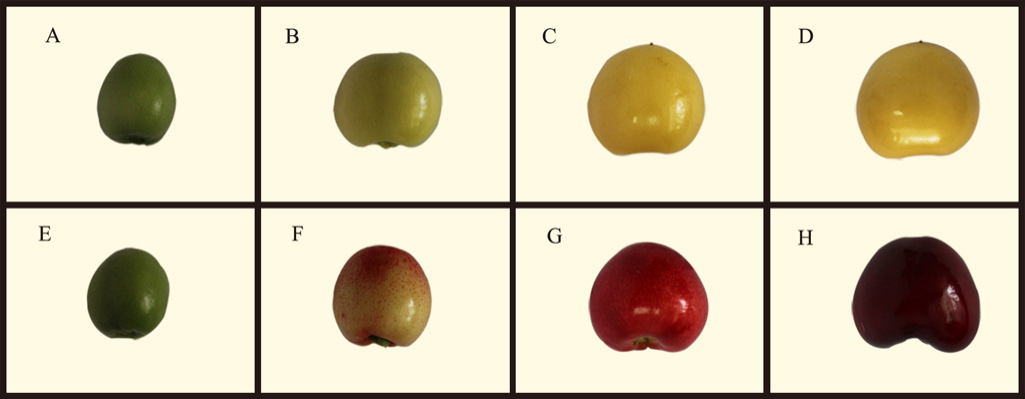
Fruits of *P*. *avium* L. ‘13–33’ and ‘Tieton’ used in deep sequencing. (A) ‘13–33’ fruit at 20 DAF (stage 1). (B) ‘13–33’ fruit at 35 DAF (stage 2). (C) ‘13–33’ fruit at 45 DAF (stage 3). (D) ‘13–33’ fruit at 55 DAF (stage 4). (E) ‘Tieton’ fruit at 20 DAF (stage 1). (F) ‘Tieton’ fruit at 35 DAF (stage 2). (G) ‘Tieton’ fruit at 45 DAF (stage 3). (H) ‘Tieton’ fruit at 55 DAF (stage 4).

The pure yellow sweet cherry cultivar *P*. *avium* L. (‘13–33’) and the red sweet cherry cultivar *P*. *avium* L. (‘Tieton’) were used in this study [32]. The plants were grown in Shandong Institute of Pomology, Tai’an, Shandong Province, China. According to the data from our laboratory, the fruit development periods of the two cultivars were similar. The total sugar accumulation pattern was the same in the two cultivars, beginning at the early stages and accelerating during ripening. The total acid content first increased and then decreased until the fruit was ripe in both cultivars (S1 Table). Fruit samples were collected at four different ripening stages: 20 days after flowering (DAF) (stage 1), 35 DAF (stage 2), 45 DAF (stage 3) and 55 DAF (stage 4). Fig. 1 shows samples of fruits at the four different ripening stages. After collection, samples were flash-frozen in liquid nitrogen and stored at -80°C until further processing.

The total anthocyanin content in the sweet cherry cultivars was assayed using the method presented by Wang et al [33]. Fruits were crushed and pooled to obtain three replicates. Anthocyanins were extracted in 10 ml of methanol (containing 1% hydrochloric acid) for 2 h at 4°C in darkness. Anthocyanin content was measured at 553 and 600 nm.

### Total RNA extraction

Total RNA was extracted using a modified CTAB method [34]. RNA quantity and quality (purity and integrity) were analyzed using a NanoPhotometer spectrophotometer (IMPLEN, Westlake Village, CA, USA) and an Agilent Bioanalyzer 2100 system (Agilent Technologies, CA, USA), respectively.

### Library construction and transcriptome sequencing

Total RNA from 4 different fruit ripening stages of the yellow (YTR) and red (RTR) cultivars was pooled prior to library preparation in the two experimental groups. Equimolar quantities of total RNA from samples at each stage were combined into one pool. Prior to cDNA library construction, poly-T oligo-attached magnetic beads were used to purify the mRNA, which was then broken into short fragments of approximately 200 bp. The fragments were used to synthesize first-strand cDNA using random oligonucleotides and SuperScript II. Second-strand cDNA was then synthesized using DNA polymerase I and RNase H. The double-stranded cDNA fragments were subjected to end repair, and sequencing adapters were ligated to both ends. The final cDNA library was selectively enriched by PCR and purified using the AMPure XP system (Beckman Coulter, Beverly, USA). The library preparations were sequenced by Novogene Bioinformatics Technology Co., Ltd (Beijing, China) on an Illumina HiSeq 2000 platform. Then, 100-bp paired-end reads were generated, and all raw sequence read data were deposited in the NCBI Short Read Archive (SRA) database under accession number SRP044388.

### *De novo* transcriptome assembly

The raw reads were first processed through in-house Perl scripts, which removed reads containing the sequencing adapters, reads with over 10% ambiguous ‘N’ nucleotides, and reads with over 50% of bases with a quality score lower than 5. The remaining clean reads from each library were assembled using a combined *de novo* transcriptome assembly strategy. *De novo* assembly of transcripts was performed using Trinity software as previously described for *de novo* transcriptome assembly without a reference genome [35].

### Functional annotation and classification

Unigene function was annotated using NCBI BLAST 2.2.28^+^ with an E-value threshold of 10^-5^ for the NCBI non-redundant protein (Nr) database, the NCBI non-redundant nucleotide sequences database (Nt), the Swiss-Prot protein database, and the euKaryotic Ortholog Groups of proteins (KOG) database. Protein family (Pfam) was assigned using the HMMER 3.0 package. The Kyoto Encyclopedia of Genes and Genomes (KEGG) Ortholog (KO) database categories were assigned to the unigene sequences using the KEGG Automatic Annotation Server (KAAS) online [36]. Blast2GO v2.5 was used to obtain Gene Ontology (GO) annotation of unigenes based on BLASTX hits against the Nr database with a cut-off E-value of 10^-5^ [37].

### Digital gene expression sequencing and mapping to reference transcripts

The eight DGE libraries prepared from the four different ripening stage samples (R1 and Y1: 20 DAF fruits, R2 and Y2: 35 DAF fruits, R3 and Y3: 45 DAF fruits and R4 and Y4: 55 DAF fruits) were constructed using the Illumina TruSeq RNA Sample Preparation Kit. The library preparations were sequenced on the Illumina HiSeq 2000 platform. The raw reads were deposited in the NCBI SRA database (accession number SRP044388).

To map the DGE reads, clean reads were obtained by removing low-quality reads and reads containing adapters or poly-N stretches from the raw data. The clean reads were mapped back onto the assembled transcriptome for each sample using RSEM [38].

### Analysis of differential gene expression

For gene expression analysis, the read numbers mapped to each gene were counted using HTSeq v0.5.4p3 and then normalized to RPKM (Reads per Kilobase per Million mapped reads) [39]. The DESeq R package (1.10.1) was used for differential expression analysis of the unigenes between two samples. The differentially expressed genes (DEGs) were filtered for a corrected P-value threshold of 0.005 (P values were adjusted using the Benjamini & Hochberg method) and log2 (fold change) of 1. We used GOseq (1.10.0) for GO enrichment analysis of all differentially expressed genes [40]. KEGG enrichment analysis was performed using the KOBAS software [41].

### qRT-PCR analysis

Eighteen unigenes were chosen for validation using qRT-PCR. Specific primer pairs for selected genes used in qRT-PCR were designed as shown in S2 Table. The cDNA was transcribed from 1 μg of total RNA using the Thermo Scientific Revertaid First Strand cDNA Synthesis Kit (Thermo, USA) in 20 μL of reaction mixture. The qRT-PCR was performed with the ABI 7500 Fast Real-Time Detection System (Applied Biosystems) with the Ultra SYBR Mix (with ROX) (CWBIO, Beijing, China). The thermal profile for SYBR Green I RT-PCR was 95°C for 10 min, followed by 40 cycles of 95°C for 15 s and 55°C for 1 min. Each plate was repeated three times in independent runs for all reference and selected genes. The reference gene (*β-ACTIN*) was used for normalization. The comparative CT method (2^-ΔΔ^CT method) was used to analyze the expression levels of the different genes [42].

### Statistical analysis

All of the experiments analyzed using data comparisons were repeated three times. Statistical analyses were performed using variance (ANOVA) followed by Duncan’s new multiple range tests with SPSS version 16.0 (SPSS, Chicago, IL, USA). A significance level of p < 0.05 was applied.

## Results and Discussion

### RNA-Seq and *de novo* transcriptome assembly

Two cDNA libraries, YTR and RTR, were constructed from the total RNA of the yellow (YTR) and red (RTR) cultivars’ fruit. Fruit were collected at 20 DAF (stage 1, Fig. 1A, 1E), 35 DAF (stage 2, Fig. 1B, 1F), 45 DAF (stage 3, Fig. 1C, 1G), and 55 DAF (stage 4, Fig. 1D, 1H). These cDNA libraries were subjected to pair-end reading with the Illumina HiSeq 2000 platform, generating 33,566,702 and 35,223,154 paired-end raw reads of 100 bp in length, respectively (Table 1). All of the raw reads are available in the NCBI SRA database (accession number SRP044388). After removing the low-quality reads and trimming the adapter sequences, 32,398,690 and 33,993,880 clean reads were obtained for the YTR and RTR libraries, respectively.

**Table 1:** Summary of the sequencing and *de novo* assembly.

Sequences	YTR	RTR
**Before trimming**		
Total nucleotides (bp)	6,713,340,400	7,044,750,800
Number of raw reads	33,566,702	35,223,154
**After trimming**		
Number of clean reads	32,398,690	33,993,880
GC content (%)	45.73	45.99
Q20 percentage (%)	95.78	95.82
**After assembly**		
Number of transcripts of combined data	81,670	
Number of unigenes of combined data	43,128	
Total nucleotides (nt) of transcripts (bp)	110,084,099	
Total nucleotides (nt) of unigenes (bp)	40,691,890	
Mean length of transcripts (bp)	1,348	
Mean length of unigenes (bp)	944	
N50 of unigenes (bp)	1,781	
N90 of unigenes (bp)	344	

The clean reads from the two libraries were assembled into 81,670 transcripts with an average length of 1348 bp using the Trinity software, and 43,128 unigenes were obtained (Table 1). Assembled unigenes ranged from 201 bp to 15,614 bp, and approximately half of them (22,281; 51.67%) were 200–500 bp in length (S1 Fig.). Compared to the sweet cherry transcriptome analysis published in 2014, this study was superior based on the N50 value (1781 bp), the average length of unigene (944 bp) obtained and the accuracy of the assembled results using paired-end sequencing [31]. The sequencing and assembly results suggest that the unigene data were highly reliable for further analysis.

### Functional annotation and classification

**Fig 2:**
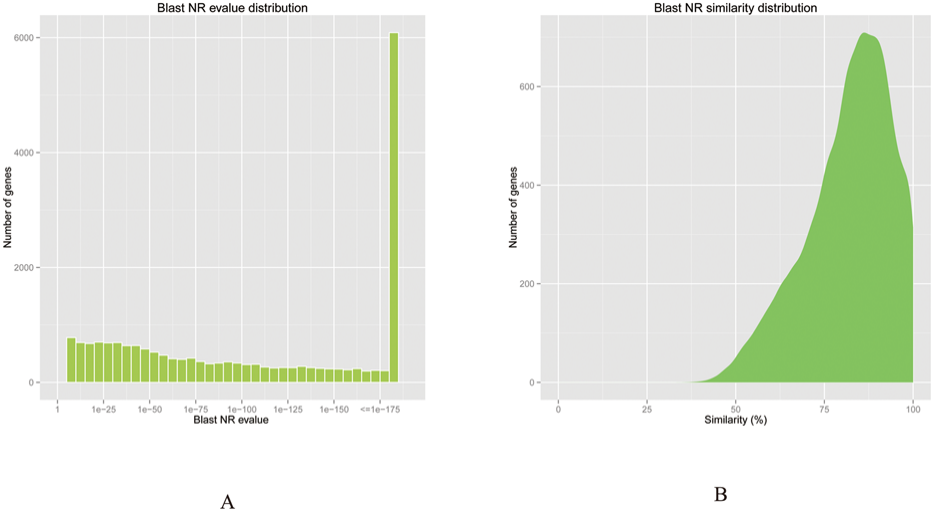
*E*-value and similarity distribution of sweet cherry fruit tissue transcriptome unigenes with annotation to the Nr database. (A) *E*-value distribution of annotated unigenes; (B) Similarity distribution of annotated unigenes.

For functional annotation of the sweet cherry fruit transcriptome, 22,452 unigenes were BLASTed against seven public databases (Table 2). Approximately 20,095 (46.59%) and 14,154 (32.81%) unigenes showed significant BLAST hits against known sequences in the Nr and Nt databases, respectively. A total of 70.22% (14,111) of the matched sequences showed high homology, with an E-value < IE-50, and 63.7% (12,801) of the matched sequences with a similarity value of 80% in the Nr database (Fig. 2). Additionally, 15,394 (35.69%), 8,645 (20.04%), 15,345 (35.58%), 4,035 (9.35%) and 16,853 (39.07%) unigenes were annotated in the Swiss-Prot, KOG, Pfam, KO and GO databases, respectively (Table 2). Due to limitations in the genomic and expressed sequence tag (EST) information available for *P*. *avium* L., only 52.05% of the total unigenes were annotated from at least one database.

**Table 2:** Summary of the annotations for the assembled sweet cherry unigenes in public databases.

Database	Number of annotated unigenes	Percentage of annotated unigenes (%)
Nr	20,095	46.59
Nt	14,154	32.81
KO	4,035	9.35
Swiss-Prot	15,394	35.69
PFAM	15,345	35.58
GO	16,853	39.07
KOG	8,645	20.04
Annotated in all databases	2,246	5.2
Annotated in at least one database	22,452	52.05

**Fig 3:**
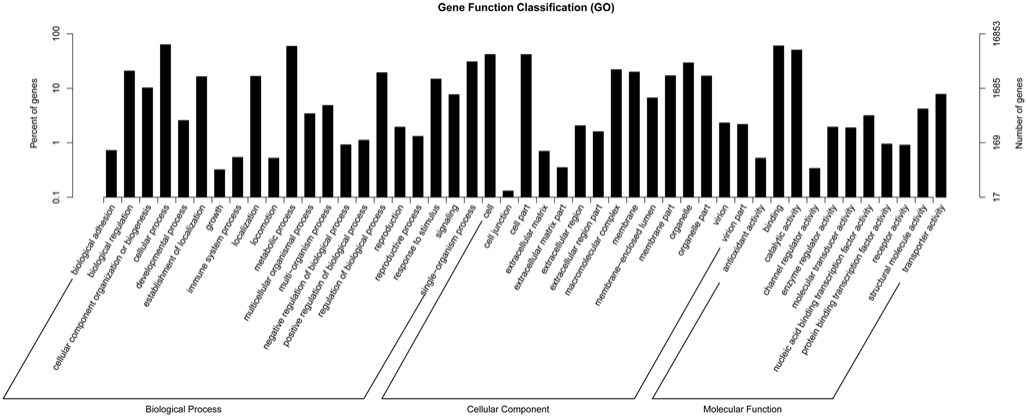
GO classification of unigenes of *P*. *avium* L fruit. The results are summarized in three main GO categories: Biological process, Cellular Component and Molecular Function.

Gene Ontology (GO), an international standardized gene function classification system, was used to classify the function of the predicted *P*. *avium* L. genes. Sequence homology revealed that 16,853 of the assembled unigenes were assigned at least one GO term (Table 2). Furthermore, 46,748 unigenes were assigned to the Biological Process category, 34,527 unigenes were assigned to the Cellular Component category, and 22,307 unigenes were assigned to the Molecular Function category. These unigenes were further classified into 55 functional subcategories (Fig. 3). The most common assignments in the Biological Process category were cellular process (10,654 unigenes, 22.79%), metabolic process (9,974 unigenes, 21.34%) and single-organism process (5,180 unigenes, 11.08%). In the Cellular Component category, the majority of unigenes were grouped into the cell (7,049 unigenes, 20.42%), cell part (7,037 unigenes, 20.38%) and organelle (4,945 unigenes, 14.32%) subcategories. Genes in the Molecular Function category were primarily sorted into the binding (10134 unigenes, 45.43%), catalytic activity (8513 unigenes, 38.16%) and macromolecular complex (3,709 unigenes, 10.74%) subcategories.

**Fig 4:**
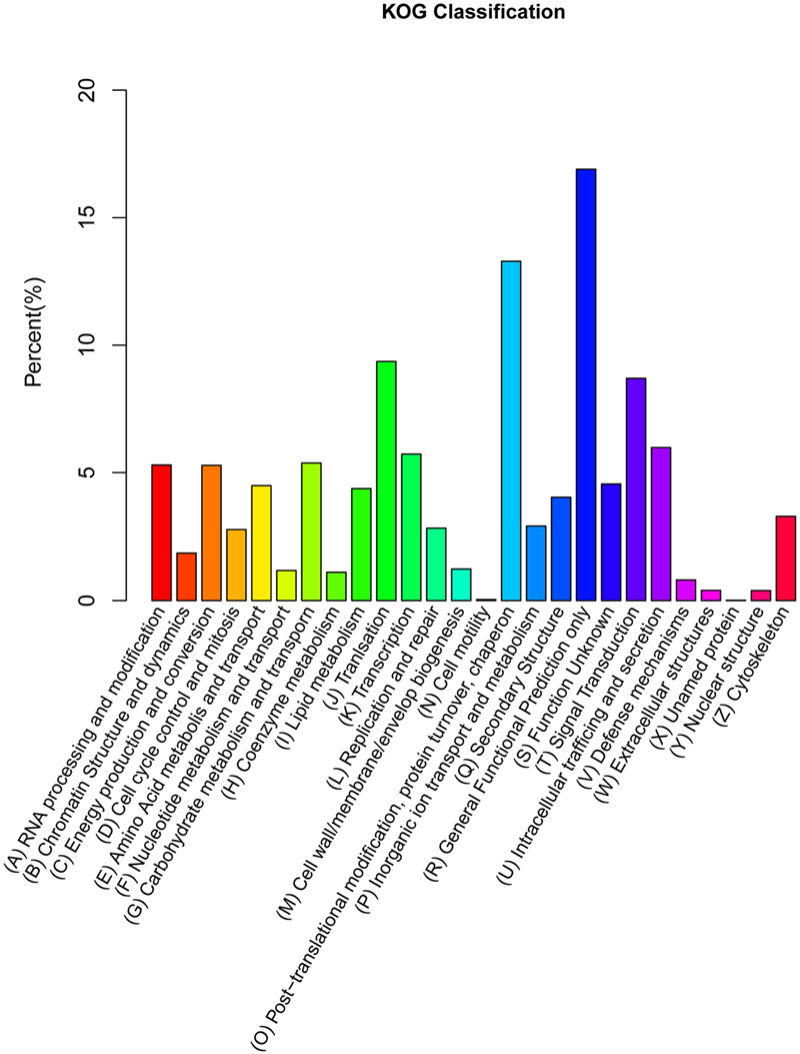
Histogram of KOG classification. The 8,645 unigenes were grouped into 25 KOG categories.

To further evaluate the completeness of our transcriptome library and the effectiveness of the annotation process, KOG annotations were selected, and 8,645 unigenes were assigned to 26 KOG categories (Fig. 4). Among the 26 KOG categories, the cluster for General Functional Prediction (1,461 unigenes, 16.90%) represented the largest group, followed by Post-translational modification, protein turnover, chaperone (1,149 unigenes, 13.29%) and Translation (809 unigenes, 9.36%). Cell motility (4 unigenes, 0.05%), Nuclear structure (34 unigenes, 0.39%) and Extracellular structures (35 unigenes, 0.40%) represented the smallest groups.

To identify the biological pathways activated in sweet cherry fruit, we mapped the annotated sequences to the canonical reference pathways in the KEGG database [36]. Significant matches were found for 4,035 unigenes, which were assigned to 242 KEGG pathways (S2 Fig.; S3 Table). In total, 2,748 unigenes were identified in metabolism, and the main metabolic pathways were Carbohydrate metabolism (564 unigenes), Energy metabolism (511 unigenes) and Amino acid metabolism (392 unigenes). From these pathways, information on sweet cherry metabolism can be obtained. We concentrated on the ‘Biosynthesis of other secondary metabolites’ category in relation to fruit pigmentation. The KEGG pathway analysis also revealed that 149 unigenes were classified into 12 subcategories within the ‘Biosynthesis of other secondary metabolites’ category. Among these, the cluster for ‘Phenylpropanoid biosynthesis’ represented the largest group, followed by ‘Flavonoid biosynthesis’ and ‘Flavone and flavonol biosynthesis’.

### Digital gene expression library sequencing and mapping

To investigate the gene expression patterns in red and yellow sweet cherry fruits during the ripening process, eight DGE libraries were constructed and sequenced using Illumina deep sequencing technology. Using Illumina sequencing, we obtained over 7.0 million raw reads in each library (Table 3). The raw reads are available at the NCBI SRA database (accession number SRP044388). Following the transformation of raw sequences into clean reads, the total number of reads per library ranged from 7,248,161 to 11,442,383, and more than 90% of the total reads in each library were clean reads. We then mapped the clean reads in each library to our transcriptome reference database, which contained 43,128 unigenes. Approximately 94.56% to 95.95% of the clean reads in each sample were mapped to our transcriptome reference database (Table 3).

**Table 3:** Statistics of DGE library sequencing and read mapping.

Library	Raw reads	Clean reads	Clean bases	Clean reads /Raw reads (%)	Total mapped reads	Total mapped reads /Clean reads (%)
Y1	7633776	7,568,503	0.76G	99.14%	7,156,815	94.56%
Y2	9016540	8,929,925	0.89G	99.04%	8,522,912	95.44%
Y3	8899691	8,821,425	0.88G	99.12%	8,453,179	95.83%
Y4	7449167	7,381,098	0.74G	99.09%	7,016,375	95.06%
R1	7311958	7,248,161	0.72G	99.13%	6,880,047	94.92%
R2	9964196	9,879,901	0.99G	99.15%	9,466,562	95.82%
R3	11541278	11,442,383	1.14G	99.14%	10,978,727	95.95%
R4	10465601	10,369,923	1.04G	99.09%	9,836,442	94.86%

Notes: Y1: ‘13–33’ fruit at 20 DAF (stage 1); Y2: ‘13–33’ fruit at 35 DAF (stage 2); Y3: ‘13–33’ fruit at 45 DAF (stage 3); Y4: ‘13–33’ fruit at 55 DAF (stage 4); R1: ‘Tieton’ fruit at 20 DAF (stage 1); R2: ‘Tieton’ fruit at 35 DAF (stage 2); R3: ‘Tieton’ fruit at 45 DAF (stage 3); R4: ‘Tieton’ fruit at 55 DAF (stage 4).

### Analysis of differential gene expression

To compare the changes in gene expression between different cultivars and different stages of ripening, we normalized the gene expression levels to the RPKM. All uniquely mapped reads were used to calculate the genes’ RPKM values. The differentially expressed genes were hierarchically clustered based on the log_10_RPKM of the eight samples, allowing us to observe the overall gene expression pattern. The red bands indicate high gene expression, and the blue bands indicate low gene expression (S3 Fig.).

**Fig 5:**
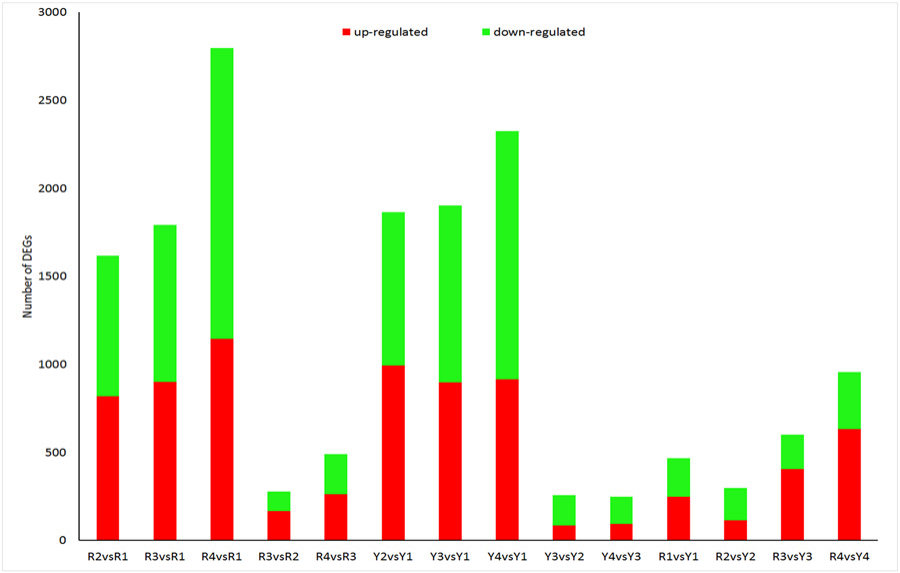
Unigenes differentially expressed between different stages of fruit development and between cultivars at the same stage. Upregulated (red) and downregulated (green) unigenes were quantified. The results of 14 comparisons between each two samples are shown.

The genes whose expression differed in the two samples were identified and filtered for corrected P values < 0.005 and log2 (fold change) values > 1. We compared the DEGs between different stages within a cultivar and between cultivars within a specific stage. The number of DEGs among these comparisons varied; approximately 250–2797 unigenes displayed significant changes in expression, and the average number was 1136 (S4 Table). The number of upregulated and downregulated unigenes are shown in Fig. 5.

To illustrate the DEGs detected in the different developmental stages of fruits from the yellow and red cultivars, we conducted GO functional enrichment and KEGG pathway analyses. The DEGs were clustered into three main categories of the GO classification: Biological Process, Cellular Function and Molecular Function (S5 Table). The genes from the different expression clusters associated with the different functional categories clearly indicate the molecular and cellular events involved in sweet cherry fruit ripening. KEGG pathway enrichment analysis of the differentially expressed genes revealed effects on various metabolic processes, particularly in starch and sucrose metabolism, the biosynthesis of secondary metabolites, and amino acid metabolism (S6 Table). The predicted biosynthesis pathways for the unigenes differed between the libraries, and most of the unigenes involved in these pathways were differentially expressed. In particular, we found that the unigenes predicted to be involved in anthocyanin biosynthesis were significantly increased or decreased in different DGE libraries.

### Genes from the sweet cherry fruit transcriptome are involved in anthocyanin biosynthesis

**Fig 6:**
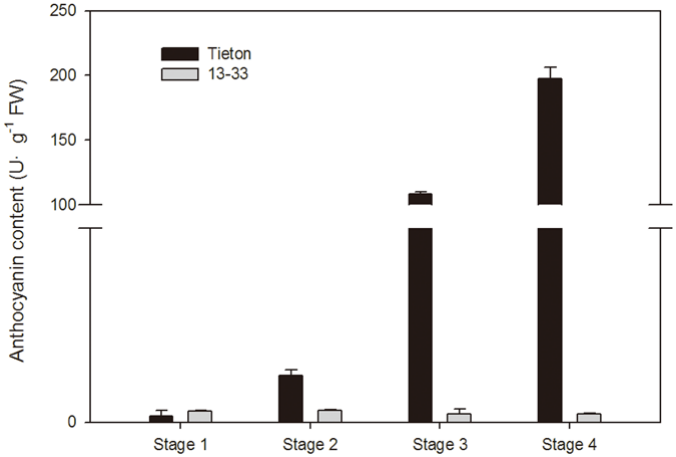
Comparison of anthocyanin content in different ripening stages of the red sweet cherry cultivar ‘Tieton’ and the yellow sweet cherry cultivar ‘13–33’.

**Fig 7:**
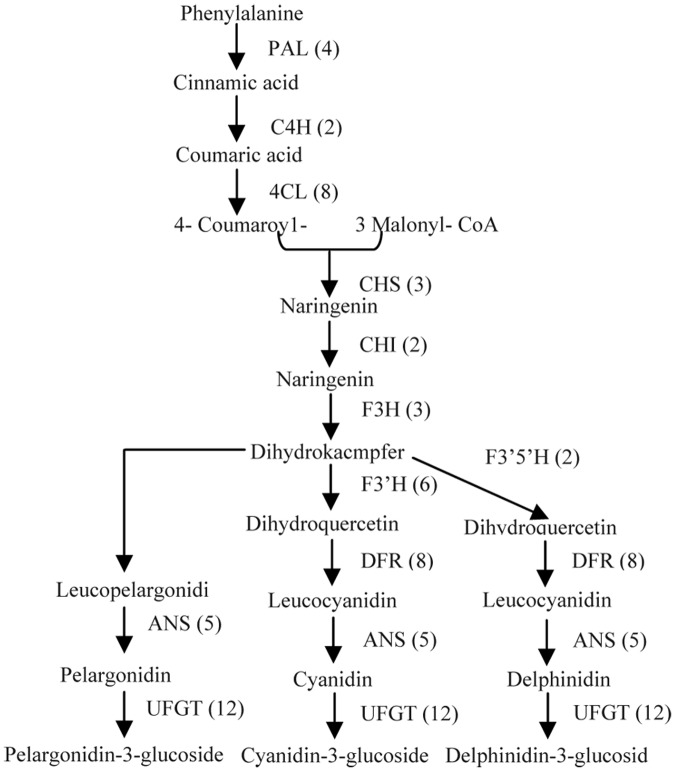
Unigenes involved in the anthocyanin biosynthesis of sweet cherry. The numbers in parentheses indicate the number of unigenes for each gene in the transcriptome library.

In the later ripening stages, sweet cherry fruit undergoes a rapid color change. The red color of sweet cherry fruit is a result of the increase in total anthocyanins, consisting mostly of cyanidin-3-rutinoside and cyanidin-3-glucoside [43, 44]. In this study, the anthocyanin content in the red sweet cherry ‘Tieton’ fruit increased from 0.67 U·g^-1^ FW to 197.4 U·g^-1^ FW as the color changed from green to dark red during ripening (Fig. 6). This accumulation pattern is comparable to previously reported trends for the red color of sweet cherry [22]. However, the anthocyanin content of the yellow sweet cherry ‘13–33’ fruit was almost unchanged and remained at a very low level during fruit ripening in our study (Fig. 6), which, to our knowledge, is the first time these data have been assessed in sweet cherry. Previous studies have shown that fruit anthocyanin content is correlated with the expression of anthocyanin biosynthetic genes in many crops, including apple, pear, and grape [17, 45, 46, 47]. In this study, unigenes participating in the anthocyanin biosynthetic pathway were selected and studied. A total of 72 unigenes encoding 11 enzymes were assigned to the anthocyanin biosynthetic pathway based on a KEGG pathway assignment (Fig. 7). More than one unigene sequence was annotated as the same enzyme, indicating that these unigenes represent different fragments of a single transcript or different members of a gene family [48]. For example, CHS is the first committed enzyme in this pathway [49]; 6 unigene sequences (including unigene comp27880_c0, comp29351_c0 and comp16012_c1, among others) from the transcriptome database were annotated as CHS.

In the green apple varieties ‘Crown’ and ‘Granny Smith’, *CHS*, *F3H*, *DFR* and *UFGT* are not expressed or are expressed at low levels; however, in the red apple varieties ‘Gala’ and ‘Cripps Red’, the expression levels of these genes are very high [50]. Previous qRT-PCR analyses have shown that the expression levels of *CHS*, *CHI*, *F3H*, *DFR* and *ANS* increased along with the anthocyanin synthesis process in a red sweet cherry cultivar but were expressed at low levels and were almost unchanged during fruit ripening in a bicolored cultivar [21, 22]. In this study, 13 candidate unigenes that are responsible for anthocyanin synthesis in fruit were identified from the DEG data including *PAL* (1 unigene), *4CL* (2 unigenes), *CHS* (2 unigenes), *CHI* (1 unigene), *F3H* (1 unigene), *F3’H* (1 unigene), *DFR* (1 unigene), *ANS* (1 unigene) and *UFGT* (3 unigenes) (Table 4). The expression of these 13 unigenes exhibited distinctive patterns in the two varieties. In fruit of the red cultivar ‘Tieton’, all of the DEGs encoding anthocyanin biosynthesis showed significantly upregulated expression during the ripening process, particularly when the fruit turned red (stage 3 and stage 4) (Table 4). This result is in agreement with previously reported qRT-PCR results [22]. In contrast, all of the DEGs encoding anthocyanin biosynthesis in fruit of the yellow cultivar ‘13–33’ showed significantly downregulated expression during stage 1 and stage 2 and remained at a low expression level in stage 3 and stage 4. These results are consistent with the accumulation of anthocyanin in these cultivars (Fig. 6, Table 4). The expression levels of *PAL*, *CHS* and *F3’H* in the red fruit were lower than those of the yellow fruit at stage 1. However, in stages 3 and 4, the expression levels of *PAL*, *4CL*, *CHS*, *CHI*, *F3H*, *DFR*, *F3’H*, *ANS*, and *UFGT* in the red fruit were higher than those of the yellow fruit. This result indicates that the biosynthesis of anthocyanin compounds are maintained at high levels in the red cultivar ‘Tieton’ when the fruit is turning red. The expression of these genes might be required for sweet cherry coloration because anthocyanin composition is primarily responsible for alterations in fruit color. Previous studies have shown that the expression of *UFGT* is critical for fruit coloration in many plants, such as grape, strawberry and lychee [51, 52, 53]. There is also a high correlation for anthocyanin accumulation in the bicolored cultivar ‘Caihong’ and the red cultivar ‘Hongdeng’ of sweet cherry [22]. However in the present study, there were no significant changes in the expression of 3 unigenes encoding *UFGT* in the yellow cultivar ‘13–33’, but a gradual increase in *UFGT* was correlated with the accumulation of anthocyanin in the red cultivar ‘Tieton’. This result indicates that *UFGT* may play a key role in sweet cherry coloration.

**Table 4:** Expression profiles of anthocyanin biosynthesis genes in sweet cherry fruit.

Gene name	Unigene ID	Gene length	RPKM							
			Stage 1		Stage 2		Stage 3		Stage 4	
			R1	Y1	R2	Y2	R3	Y3	R4	Y4
*PAL*	comp26141_c0	2,717	250.35	454.14	178.27	43.20	268.74	26.59	642.21	23.76
*4CL*	comp21677_c0	2,223	67.60	114.47	36.64	10.63	81.08	8.52	181.34	9.01
	comp24772_c0	2,065	54.80	56.05	27.27	25.67	42.20	18.88	28.01	16.60
*CHS*	comp27880_c0	1,684	257.11	864.24	124.09	7.166	431.97	18.00	1,001.81	5.155
	comp29351_c0	282	54.14	546.25	22.49	1.296	86.44	8.67	298.98	1.57
*CHI*	comp24444_c0	1,090	205.26	210.68	262.66	18.11	518.95	15.02	548.06	8.98
*F3H*	comp22775_c0	1,539	623.61	909.37	423.48	75.80	768.49	82.69	880.70	53.34
*DFR*	comp14360_c0	1,680	246.19	489.64	246.57	36.13	585.27	35.51	1,579.94	28.53
*F3’H*	comp19284_c0	2,120	120.93	279.77	125.91	21.38	265.99	19.77	545.25	15.75
*ANS*	comp28941_c0	1,680	226.22	293.52	755.24	147.66	1,507.65	245.56	1,876.1	151.16
*UFGT*	comp22538_c0	4837	25.51	21.60	160.48	18.87	246.31	16.33	734.84	19.58
	comp13406_c0	1826	28.31	24.44	25.93	7.16	35.72	6.75	66.37	28.31
	comp26856_c0	1859	18.70	25.97	21.83	42.22	36.59	35.77	82.13	18.70
	comp26856_c0	1859	18.70	25.97	21.83	42.22	36.59	35.77	82.13	18.70
*MYB*	comp26801_c1	1805	1.84	0.32	89.48	0.34	153.39	4.47	742.37	0.08
	comp33353_c0	1258	15.74	27.03	25.27	8.53	17.36	0.96	55.80	5.07
	comp20483_c0	1019	2.82	0.57	5.04	2.39	14.87	0.25	20.48	0.87
	comp2616_c0	1256	1.08	0.81	1.13	0	0.75	29.11	3.17	0
*bHLH*	comp24411_c0	2985	34.04	62.75	39.42	31.77	43.88	11.85	63.09	37.79
	comp21245_c0	2838	16.25	32.11	46.55	28.39	32.23	28.41	41.44	26.93
*WD40*	comp19899_c0	1915	46.62	28.46	38.83	41.56	37.70	0	55.46	38.98

Anthocyanin biosynthesis structural genes are controlled by transcription factors such as MYB and the basic-helix-loop-helix (b HLH) and WD40 families [54, 55, 56]. To identify regulatory factors that potentially control anthocyanin biosynthesis, candidate transcription factors were chosen from the DEG data. It has been reported that MYB transcription factors play a key role in regulating anthocyanin biosynthesis in some fruit trees [57, 58, 59]. In our study, we first identified that 4 candidate *MYB* genes among the DEGs were annotated as *R2R3-MYB* genes; the expression of these factors increased gradually with fruit ripening in red fruit but were almost unchanged in yellow fruit (Table 4). In stage 3 and stage 4, the expression of these unigenes was strongly up-regulated in ‘Tieton’ compared to ‘13–33’. Among these unigenes, comp26801_c1 was highly homologous to *P*. *avium MYB*10, which was identified as a key positive regulator in the control of anthocyanin biosynthesis in sweet cherry fruit [21, 60]. Comp2616_c0 was highly homologous to MYB11 [*Malus* x *domestica*], which has been reported to positively regulate anthocyanin synthesis in apple calluses [61]. Comp33353_c0 and comp20483_c0 were homologous to MYB111 [*Malus* x *domestica*] and a putative MYB transcription factor [*Rosa rugosa*], respectively, although the function of both of these genes has not been reported. In addition, 2 candidate *bHLH* genes were also identified from the DEGs, designated as comp24411_ c0 and comp21245_c0; the former was highly homologous to *P*. *avium TT8*, and the latter was homologous to *AtbHLH13*. The expression patterns of these 2 candidate *bHLH* genes in the two cultivars were the same as the patterns of the *MYB* family genes (Table 4). Finally, we targeted WD40 transcription factors; 1 candidate *WD40* gene (comp19899_c0) was identified among the DEGs and upregulated during fruit ripening in the red cultivar ‘Tieton’ compared to the yellow cultivar ‘13–33’ (Table 4). Comp19899_c0 was homologous to *At TTG1*, which is known to interact with GL3, EGL3 and PAP1 to regulate anthocyanin biosynthesis in *Arabidopsis thaliana* [62]. Among these candidate transcription factors, only one MYB transcription factor’s function has been studied in sweet cherry. The remaining candidates have not been reported to be involved in anthocyanin synthesis in sweet cherry. Further studies are still needed to determine whether changes in the transcription of these candidate genes is related to the regulation of anthocyanin metabolism. Furthermore, in sweet cherry, the relationship between these transcription factors and the differentially expressed structural genes remains unclear. Consequently, this issue should also be investigated further.

**Fig 8:**
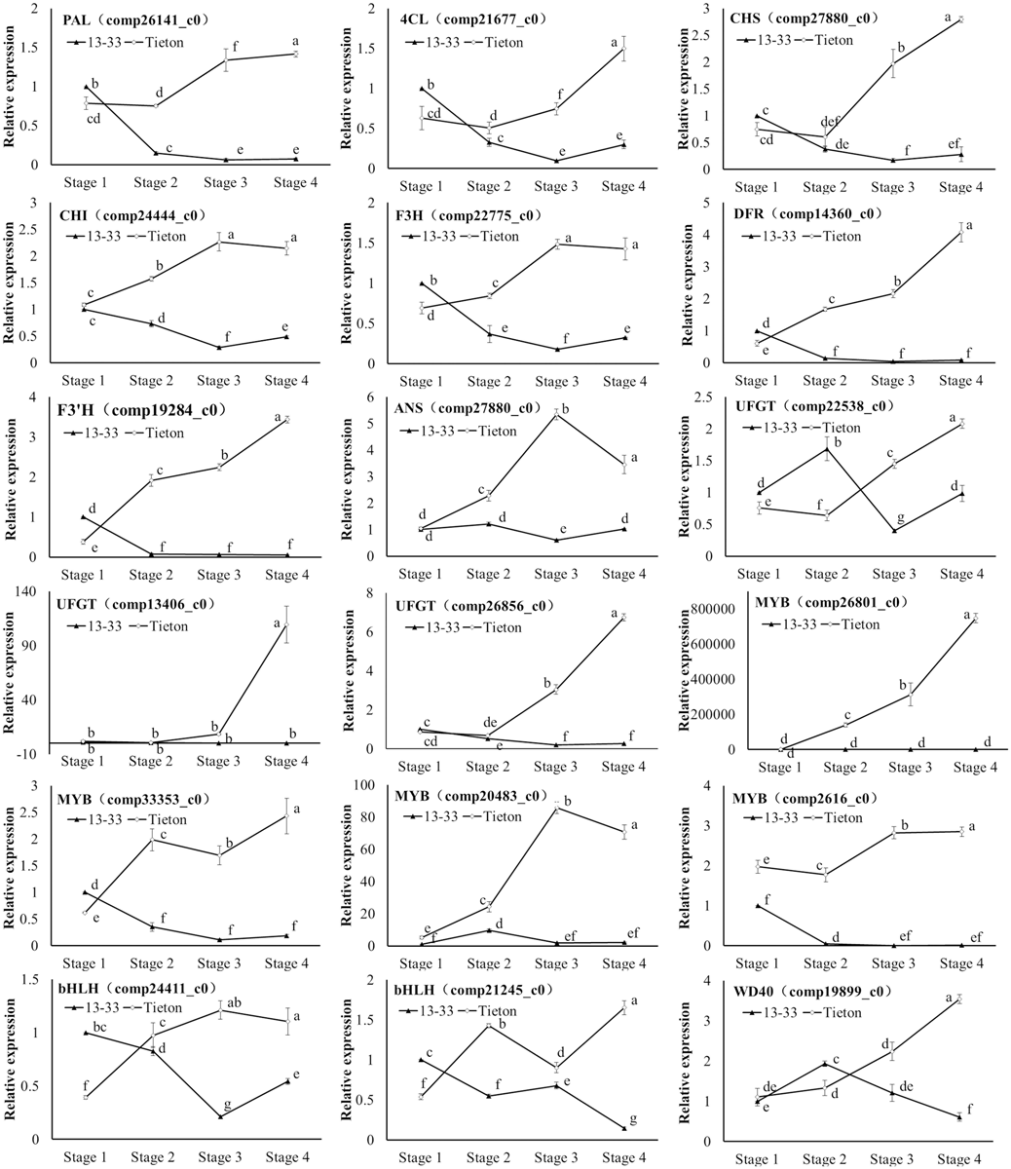
Expression analysis of 18 candidate DEGs related to anthocyanin biosynthesis in sweet cherry by qRT-PCR. The Y-axis represents the relative expression, and the X-axis depicts the fruit ripening stages. The standard error of the mean for three biological replicates (nested with three technical replicates) is represented by the error bars. Different letters on each symbol indicate statistically significant differences (P < 0.05) between two values according to ANOVA and Duncan’s new multiple range tests.

To confirm the unigenes obtained from sequencing and to further analyze the differences in the expression profiles between the red and yellow fruits during ripening, eighteen unigenes related to anthocyanin biosynthesis were chosen for qRT-PCR analysis. The red fruit showed much higher gene expression than the yellow fruit for most of the selected unigenes. These qRT-PCR results were consistent with those obtained from the DGE expression profiling (Fig. 8).

## Conclusion

This study investigated the transcriptome profiles of the fruit from red and yellow sweet cherry varieties using Illumina RNA-seq and DGE deep-sequencing technologies. This transcriptome analysis provided a total of 43,128 unigenes, of which 46.59% were annotated in the Nr database, although there is no *P*. *avium* L. reference genome sequence available. The genes that encode enzymes and transcription factors involved in anthocyanin biosynthesis were identified using *de novo* transcriptome analysis and functional annotation of novel genes, and their expression patterns were explored in the present study. The upregulated DEGs involved in anthocyanin biosynthesis might play an important role in the accumulation of anthocyanin and the development of the red color of sweet cherry. This study provides a platform for further functional genomic research on this fruit crop and a reference for studying complicated metabolic processes in non-model perennial species.

## Supporting Information

S1 Fig Histogram of sequence length distribution of the assembled unigenes in sweet cherry fruit transcriptomes. The x-axis indicates a unigene length interval from 200 bp to ≥2000 bp. The y-axis indicates the number of unigenes of each given sequence length.
(TIF)

S2 Fig KEGG classification of non-redundant unigenes of *P*. *avium* L fruit. (A) Cellular Processes; (B) Environmental Information Processing; (C) Genetic Information Processing; (D) Metabolism; (E) Organismal Systems.
(TIF)

S3 Fig Hierarchical clustering of the differentially expressed genes, using RNA-seq data derived from eight samples based on log_10_ RPKM values. Y1: ‘13–33’ fruit at 20 DAF (stage 1). Y2: ‘13–33’ fruit at 35 DAF (stage 2). Y3: ‘13–33’ fruit at 45 DAF (stage 3). Y4: ‘13–33’ fruit at 55 DAF (stage 4). R1: ‘Tieton’ fruit at 20 DAF (stage 1). R2: ‘Tieton’ fruit at 35 DAF (stage 2). R3: ‘Tieton’ fruit at 45 DAF (stage 3). R4: ‘Tieton’ fruit at 55 DAF (stage 4).
(TIF)

S1 Table Total sugar content and total acid content in the sweet cherry cultivars ‘Tieton’ and ‘13–33’. (DOC)

S2 Table Primer sequences for qRT-PCR analysis. (DOC)

S3 Table KEGG pathways and the corresponding unigene numbers and IDs in the transcriptome of sweet cherry fruit. (XLS)

S4 Table Differentially expressed genes (DEGs) and their Nr annotations in each comparative group. The DEGs were filtered with q-value < 0.005 and log2 (fold change) values > 1.
(XLS)

S5 Table Summary of the GO enrichment analysis of the DEGs in each comparison. (XLS)

S6 Table Summary of the pathway enrichment analysis of the DEGs in each comparison. (XLS)
