# Supplementary figures and images for: Comparative Transcriptome Analysis of Genes Involved in Anthocyanin Biosynthesis in the Red and Yellow Fruits of Sweet Cherry (Prunus avium L.)
Source: PLoS One. 2015 Mar 23;10(3):e0121164. doi: 10.1371/journal.pone.0121164 (PMC4370391; doi:10.1371/journal.pone.0121164)

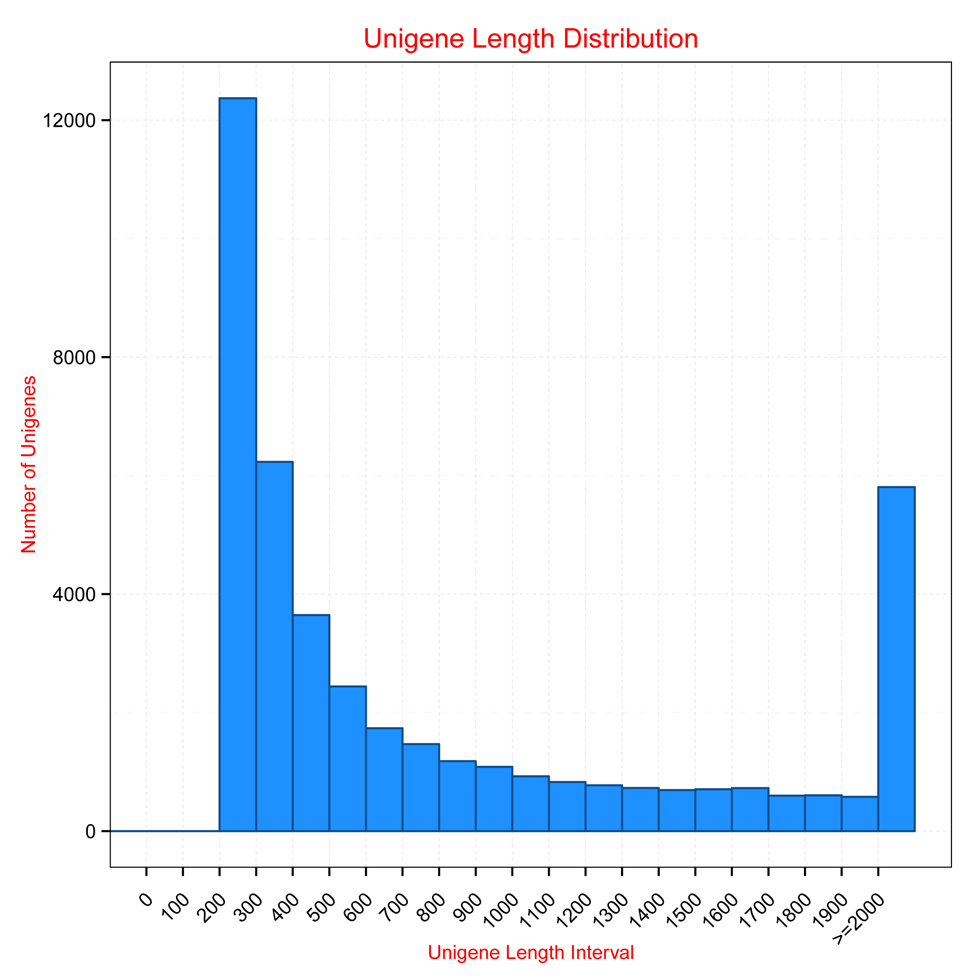

Supplement: S1 Fig — The x-axis indicates a unigene length interval from 200 bp to ≥2000 bp. The y-axis indicates the number of unigenes of each given sequence length. (TIF) [file pone.0121164.s001.tif]

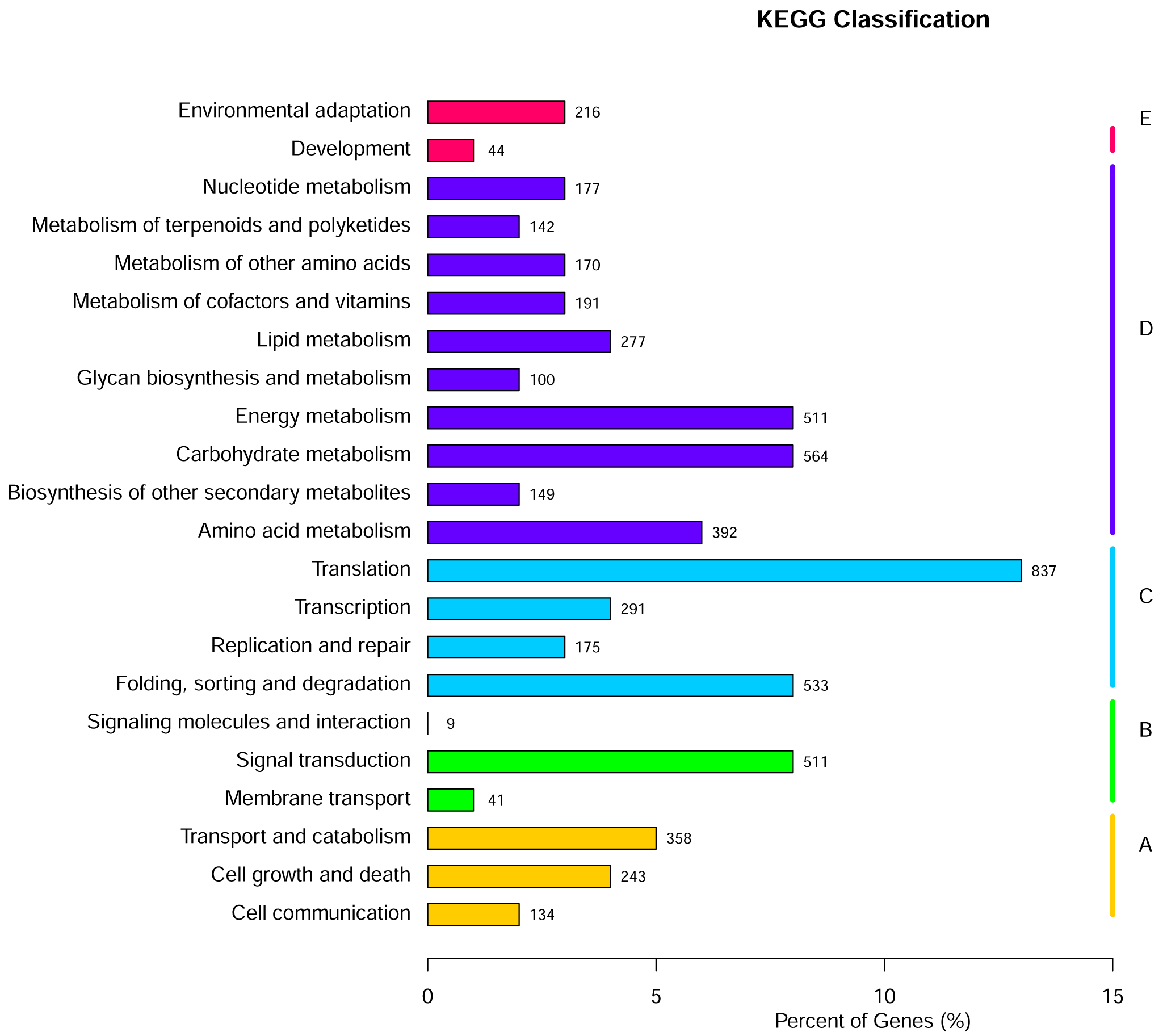

Supplement: S2 Fig — (A) Cellular Processes; (B) Environmental Information Processing; (C) Genetic Information Processing; (D) Metabolism; (E) Organismal Systems. (TIF) [file pone.0121164.s002.tif]

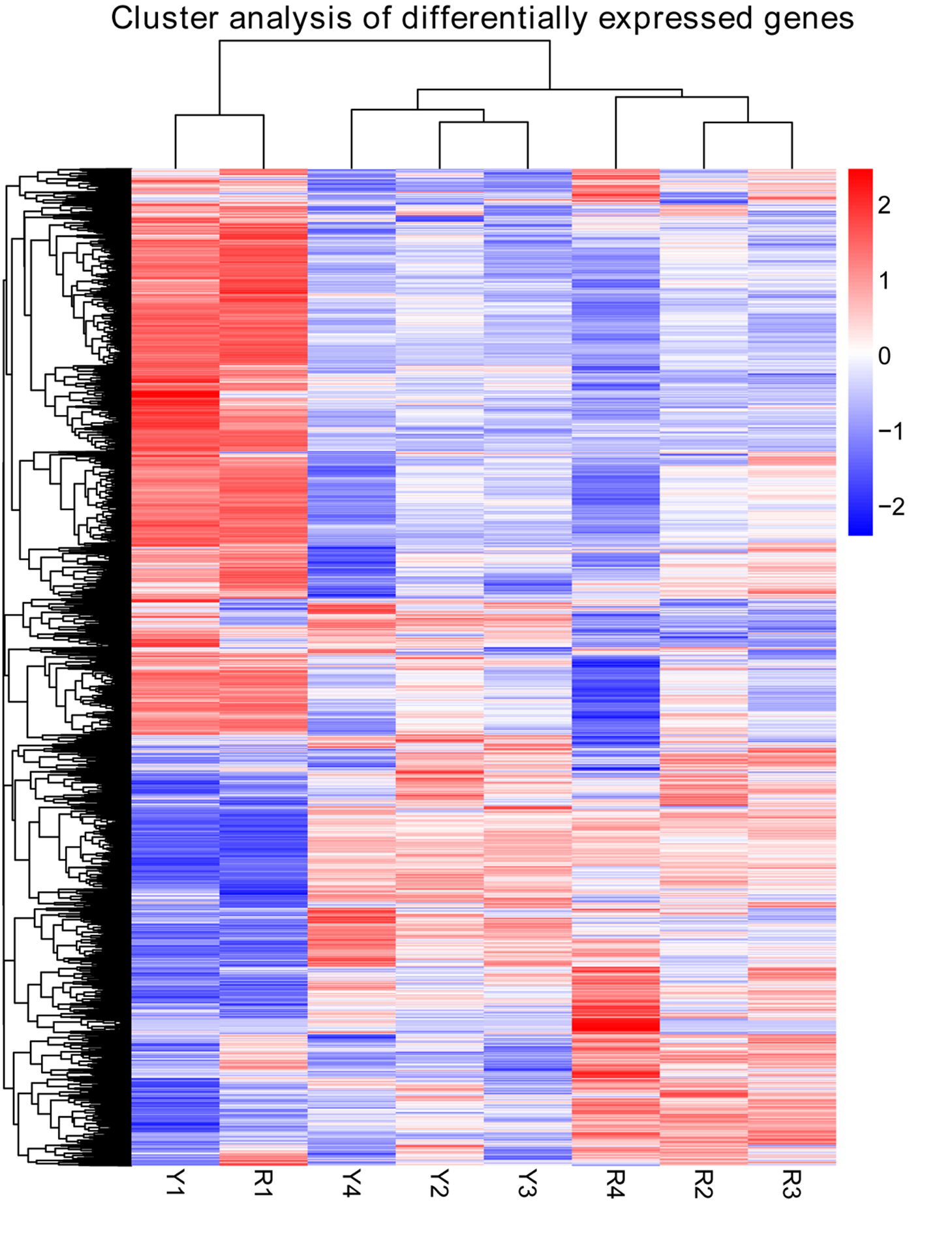

Supplement: S3 Fig — Y1: ‘13–33’ fruit at 20 DAF (stage 1). Y2: ‘13–33’ fruit at 35 DAF (stage 2). Y3: ‘13–33’ fruit at 45 DAF (stage 3). Y4: ‘13–33’ fruit at 55 DAF (stage 4). R1: ‘Tieton’ fruit at 20 DAF (stage 1). R2: ‘Tieton’ fruit at 35 DAF (stage 2). R3: ‘Tieton’ fruit at 45 DAF (stage 3). R4: ‘Tieton’ fruit at 55 DAF (stage 4). (TIF) [file pone.0121164.s003.tif]
