# Supplementary material for: Comparative Transcriptome Analysis of Genes Involved in Anthocyanin Biosynthesis in the Red and Yellow Fruits of Sweet Cherry (Prunus avium L.)
Source: PLoS One. 2015 Mar 23;10(3):e0121164. doi: 10.1371/journal.pone.0121164 (PMC4370391; doi:10.1371/journal.pone.0121164)
Supplement: S1 Table — (DOC) [file pone.0121164.s004.doc]

**S1 Table.** Total sugar content and total acid content of sweet cherry cultivars ‘Tieton’ and ’13-33’.

| Compositon | S1(20 DAF) | | S2(28 DAF) | | S3(36 DAF) | | S4(42 DAF) | | S5（50 DAF） | |
| --- | --- | --- | --- | --- | --- | --- | --- | --- | --- | --- |
|  | Tieton | 13-33 | Tieton | 13-33 | Tieton | 13-33 | Tieton | 13-33 | Tieton | 13-33 |
| Total sugar content(mg/g) | | 76.31 | | --- | | | 59.60 | | --- | | 87.89 | 67.15 | 132.29 | 172.65 | 156.29 | 238.73 | 258.66 | 302.91 |
| Total acid content(mg/g) | 4.39 | | 5.14 | | --- | | | 8.81 | | --- | | 6.77 | 10.57 | 8.92 | 8.98 | 5.56 | 6.12 | 4.78 |
