# Supplementary material for: Comparative Transcriptome Analysis of Genes Involved in Anthocyanin Biosynthesis in the Red and Yellow Fruits of Sweet Cherry (Prunus avium L.)
Source: PLoS One. 2015 Mar 23;10(3):e0121164. doi: 10.1371/journal.pone.0121164 (PMC4370391; doi:10.1371/journal.pone.0121164)
Supplement: S2 Table — (DOC) [file pone.0121164.s005.doc]

**S2 Table.** Primer sequences for qRT-PCR analysis.

| Genes | Transcripts ID | Forward primer | Reverse primer |
| --- | --- | --- | --- |
| *PAL* | comp26141_c0 | CTATGGACAATACTCGTT | GCTCAGAACAATAAGATG |
| *4CL* | comp21677_c0 | CGACGGAATTATTAGAGG | CTCATCACCATCATCATT |
| *CHS* | comp27880_c0 | GGTGCTCGTGTTCTTGTTGTG | ACTGTCGGGAAGGATGGTTTG |
| *CHI* | comp24444_c0 | TCCACCGTCAGTCAAACCACC | TCCAGCCCCCTCACCCCT |
| *F3H* | comp22775_c0 | GATTGTGGAGGCTTGTGAGGAT | GTAAATGGCTGGAGACGATGAA |
| *DFR* | comp14360_c0 | CGAAGTGACCAAGCCAACAATAA | GCAGAGGATGTAAACACCAGC |
| *F3’H* | comp19284_c0 | TAACTGAATTGGACCTAC | CATACATTGACTAGGAGAG |
| *ANS* | comp28941_c0 | GGGATTGGAAGAAGGGAGGC | ATTTGGAACACACTTGGCAGAG |
| *UFGT* | comp22538_c0 | CAGATTCCGATGATTGAA | ATTCTAATGTTGAGGCTAAT |
|  | comp13406_c0 | CCAAGGAAGAATATGAAGA | GAAGGACAGTGATATAAGG |
|  | comp26856_c0 | TAATACCGTCATCACCATCA | TGTCCAACCACTCTAAGC |
| *MYB* | comp26801_c1 | CTGCTAACATACAACGAT | TTCAACCACCTTAGTCTA |
|  | comp33353_c0 | GGTGAAGAACTATTGGAA | TTAGCCTATGGTTATTGG |
|  | comp20483_c0 | TCACTTACATTCAACAGTA | AATTGCTGACCATCTATT |
|  | comp2616_c0 | CGATGGTTGAACTATCTAA | TGTTCCAGTAATTCTTGAT |
| *bHLH* | comp24411_c0 | ATTGCGTAAGAAGATTCA | TACTACTTGACCTGTGTA |
|  | comp21245_c0 | AGACGATGAGGAAGATAG | CCAAGAGCACCATTATAC |
| *WD40* | comp19899_c0 | CATTCTACCATTATCTACGA | AACTTTATTGCTGTCCAT |
| *β-ACTIN* | | TTGTGCTGGACTCTGGTGATG | GCTCAGCAGTGGTGGTGAAC |
